# Evaluation of Vitamin D in the serum of in-hospital patients with psychosis. Retrospective study.

**DOI:** 10.1192/j.eurpsy.2023.2233

**Published:** 2023-07-19

**Authors:** E. Kapiris, E. Laskos

**Affiliations:** 1 Siggreion Psychiatric Department; 2 Μicrobiological biochemistry laboratory, Psychiatric Hospital of Athens “Dromokaiteion”, Athens, Greece

## Abstract

**Introduction:**

The reduction of vitamin D (VitD) has often been associated with pathological cognitive processes and in general with various mental illnesses^2,3^. More frequent reports of reduced concentrations of VitD concern patients with schizophrenia, however it has not been clarified whether this concerns the pathology itself of the disorder or if nutritional factors are involved^1^.

**Objectives:**

The measurement of VitD in the serum of hospitalized patients with mental illness (schizophrenia) compared to the levels of people without mental illness.

**Methods:**

The serum levels of VitD were measured in the serum of 45 psychiatric patients of psychiatric hospital “Dromokaiteion” (22 men and 23 women) mean age 59 ±14 years. The control group consisted of 49 healthy subjects (24 men and 25 women) with a mean age of 57 ±14 years (Table 1). Serum VitD levels were measured on the Architect ci4100 immunobiochemical analyzer, Abbott Laboratories Ltd, by the chemiluminescent microparticle immune assay (CMIA) method and according to the manufacturer’s instructions. The statistical analysis of the data was done with the software program SPSS V.25.

**Results:**

Mean values of Vit D (ng/ml) were 15.8±10.7 and 15.3±12.5 in male and female patients, respectively. For the control group the mean values were 22.4±7.9 in men and 26.4±13.9 in women. Vit D values in the psychiatric patients of both groups compared to the control group were statistically significantly different (men p=0.021 and women p=0.006). (Table2, 3).

**Image:**

**Figure:**
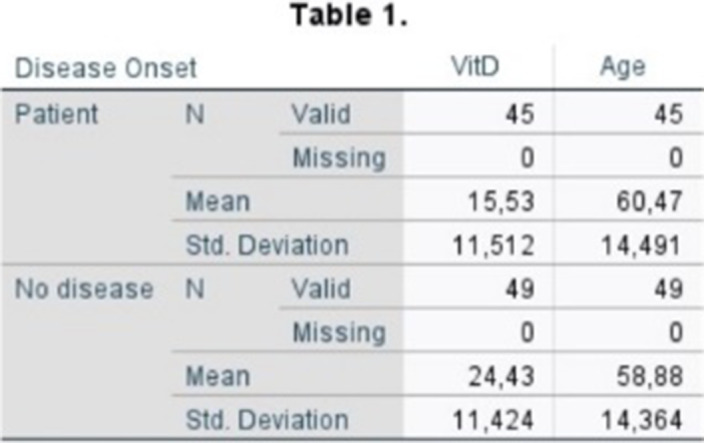

**Image 2:**

**Figure:**
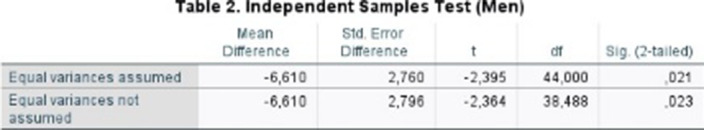

**Image 3:**

**Figure:**
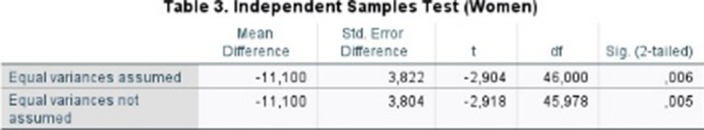

**Conclusions:**

The findings of the study are consistent with those of similar studies confirming low concentrations of VitD in the serum of patients with mental illness. This parameter should be taken into account as its measurement is not included in the routine laboratory control to date. Further future studies should correlate VitD deficiency with specific demographic and clinical characteristics.

1. Belvederi Murri M, Respino M, Masotti M, et al. Vitamin D and psychosis: mini meta-analysis. *Schizophr Res.* 2013;150(1):235-239. doi:10.1016/j.schres.2013.07.017

2. Kalueff A, Minasyan A, Keisala T, Kuuslahti M, Miettinen S, Tuohimaa P. The vitamin D neuroendocrine system as a target for novel neurotropic drugs. *CNS Neurol Disord Drug Targets.* (2006) 5:363–71. doi: 10.2174/187152706784111506

3. Oudshoorn C, Mattace-Raso FU, van der Velde N, Colin EM, van der Cammen TJ. Higher serum vitamin D3 levels are associated with better cognitive test performance in patients with Alzheimer’s disease. *Dement Geriatr Cogn Disord.* (2008) 25:539–43. doi: 10.1159/000134382

**Disclosure of Interest:**

None Declared

